# Ultrasound assessment of diaphragmatic function during weaning and after extubation in preterm newborns: brief report

**DOI:** 10.1007/s00431-026-06866-x

**Published:** 2026-03-25

**Authors:** Marcos Giovanni Santos Carvalho, Juliana Nasu Tomiyama, Marcelo Azeredo Terra, Fernanda Cordoba Lanza

**Affiliations:** 1 Graduate Program in Rehabilitation Science, Physical Therapy Department, Universidade Federal de Minas Gerais, Rua Engenho Grande, 75 Ap 9091 Bloco 2, Belo Horizonte, Minas Gerais 31320-470 Brazil; 2 Maternidade Balbina Mestrinho, Secretaria de Estado de Saúde (SES), Manaus, Amazonas Brazil; 3 Maternidade Dr. Moura Tapajoz, Secretaria Municipal de Saúde (SEMSA), Manaus, Amazonas Brazil; 4 Mterra Ensino e Consultoria, Rio de Janeiro, Rio de Janeiro Brazil

**Keywords:** Diaphragm ultrasound, Spontaneous breathing trial, Premature newborns, Endotracheal extubation, Weaning

## Abstract

To describe and assess diaphragmatic function by ultrasound during the weaning and post-extubation phases in preterm newborns. This cross-sectional study included intubated preterm newborns eligible for extubation. Inspiratory diaphragmatic thickness (IDT), expiratory diaphragmatic thickness (EDT), diaphragmatic thickening fraction (DTF), and diaphragmatic excursion (DE) were evaluated at three time points: T1—during invasive mechanical ventilation (IMV); T2—during the spontaneous breathing trial (SBT); and T3—during nasal intermittent positive pressure ventilation (NIPPV). Friedman test and Wilcoxon signed-rank post hoc test were used for comparisons (*p* < 0.05). Fifty preterm newborns were evaluated. Forty-five (mean gestational age 30.9 ± 2.2 weeks; mean weight 1377.5 ± 432 g) received IMV for 4 ± 3 days and were successfully extubated (extubation failure rate 10%). Due to the small number of failures (*n* = 5), only descriptive analyses were performed. In successfully extubated newborns, IDT and DE increased at T2 and T3 compared to T1 (*p* < 0.05), with IDT highest at T2. No significant changes were observed in EDT or DTF, which remained consistently elevated.

*Conclusion*: In preterm newborns successfully extubated, diaphragmatic thickness and excursion increased during the SBT and remained elevated after extubation. These findings may support the use of diaphragmatic ultrasound to guide extubation readiness and monitor post-extubation adaptation in preterm infants.

**Supplementary Information:**

The online version contains supplementary material available at 10.1007/s00431-026-06866-x.

**Table:** 

**What is Known:** • *Extubation decisions in preterm infants rely mainly on clinical judgment*. • *Diaphragmatic ultrasound is used to assess diaphragmatic function in various neonatal clinical situations*.		
**What is New:** • *This study provides descriptive data on diaphragmatic function throughout the entire weaning and post-extubation process in preterm newborns undergoing a spontaneous breathing trial (SBT)*. • *In successfully extubated preterm newborns, diaphragmatic thickness and excursion increased during the SBT and remained elevated after extubation, suggesting that the SBT may increase respiratory effort without reliably indicating extubation readiness in this population*.		

## Introduction

Specific health conditions and anatomical immaturity predisposes newborns, particularly preterm infants, to increased respiratory effort and respiratory failure, often requiring invasive mechanical ventilation [1, 2]. Because this support is associated with short- and long-term complications, extubation should be performed as early as clinically feasible [2, 3]. Conversely, premature extubation increases the risk of respiratory failure and reintubation, leading to higher neonatal morbidity and mortality [2]. Thus, early and successful extubation remains a major clinical goal [4].

Extubation decisions in preterm newborns are still largely based on clinical judgment [2], as commonly used clinical and physiological parameters during invasive ventilation may not reliably predict post-extubation respiratory performance. Although spontaneous breathing trials (SBT) with endotracheal continuous positive airway pressure (CPAP) are widely used in neonatal intensive care units, their predictive accuracy remains limited, likely due to the low discriminative value of the assessed variables [2, 4, 5].

Extubation failure within 48 h is commonly related to pulmonary immaturity, reduced lung aeration, and upper airway obstruction, combined with respiratory muscle immaturity and reduced fatigue resistance, all of which increase the work of breathing and predispose newborns to respiratory distress [1, 5].

Diaphragmatic ultrasound has emerged as a useful bedside tool for assessing diaphragmatic function in neonatal care [6, 7]. However, few studies have evaluated diaphragmatic parameters throughout the weaning process and after extubation. Ultrasound assessment is feasible, noninvasive, radiation-free, and easily performed by multidisciplinary teams [6, 7]. Diaphragmatic function is assessed by inspiratory and expiratory thickness (IDT, EDT), diaphragmatic thickening fraction (DTF), and diaphragmatic excursion (DE) [6, 7]. In this context, ultrasound-based assessment of diaphragmatic function may help identify preterm newborns ready for successful extubation. This study aimed to describe diaphragmatic function assessed by ultrasound during weaning and post-extubation in preterm newborns.

## Methods

### Study type and location

The study is a cross-sectional, conducted in the neonatal unit of a public maternity hospital in Manaus, AM, Brazil, between January 2022 and December 2024. The study adhered to the Strengthening the Reporting of Observational Studies in Epidemiology (STROBE) guidelines for observational research [8] (Supplementary Materials).

### Ethical aspects

The study was approved by the Research Ethics Committee of Federal University of Minas Gerais (CAAE: 59,130,222.7.0000.5149). Written informed consent was obtained from parents or legal guardians prior to enrollment. No financial or material compensation was provided. This study was conducted in accordance with the recently amended Declaration of Helsinki of 1975.

Inclusion and exclusion criteria and clinical features are described in the Supplementary Materials.

### Procedures

Newborns underwent ultrasound assessments of diaphragmatic function at three time points:

- T1—during IMV with minimal settings, 30 min before the SBT.
- T2—during the SBT (endotracheal CPAP), five minutes after initiation;
- T3—during nasal intermittent positive pressure ventilation (NIPPV), one hour post-extubation.

After the SBT, infants were reconnected to IMV with baseline settings. Extubation was performed by physiotherapists within 30 min, following protocol. All newborns received NIPPV via prongs for at least 48 h post-extubation.

Extubation criteria and the spontaneous breathing test (SBT) are provided in the Supplementary Materials.

### Ultrasound assessment

Diaphragmatic ultrasound assessments were performed by a physiotherapist experienced in image acquisition and analysis, using a VIVID system (GE Medical Systems) with a high-frequency linear transducer (8–13 MHz). Exams were conducted with neonates in the supine position, during calm breathing, approximately 1 h after feeding. To ensure comfort, the gel was pre-warmed, 1 mL of a 25% glucose solution was administered orally to keep the infants calm, and an assistant was present to soothe them. Ultrasound was used to evaluate diaphragmatic function, including IDT, EDT, DTF, and DE. The procedures for these measurements and the extubation failure criteria are provided in the Supplementary Materials.

### Statistical analysis

Continuous variables were expressed as mean ± standard deviation or median (interquartile range), depending on data distribution assessed by the Shapiro–Wilk test. Categorical variables were presented as absolute and relative frequencies. Friedman test was used to compare ultrasoung variables between three times, and Wilcoxon signed-rank post hoc test. Statistical analyses were performed using SPSS (version 24.0), with significance set at *p* < 0.05.

## Results

A total of 60 preterm infants who received invasive mechanical ventilation (IMV) for ≥ 24 h in the neonatal intensive care unit were initially enrolled. After excluding infants with hemodynamically significant congenital heart disease (*n* = 5), intracranial hemorrhage > grade III (*n* = 3), and clinical and/or hemodynamic instability leading to postponement of extubation (*n* = 2), 50 infants were included in the final analysis. All infants completed the SBT; extubation failure occurred in 5 (10%), whereas 45 were successfully extubated. Demographic and clinical characteristics are presented in Table 1, and mechanical ventilation parameters are provided in the supplementary material.

**Table 1:** Demographic characteristics of preterm newborns participating in the study (*n* = 50)

Variable	Successfully extubated *n* = 45	Extubation failure *n* = 5
Postmenstrual age (weeks)	30.9 ± 2.2	29.6 ± 2.5
Weight at study (grams)	1377 ± 432	1179 ± 342
Cardiorespiratory parameters		
Heart rate (beats/min)	156 ± 15	166 ± 15
Respiratory rate (breaths/min)	45 ± 10	43 ± 9
Oxygen saturation (%)	97 ± 2	97 ± 1
Female sex (%)	51	20
Antenatal steroid therapy (%)	57.8	60
Cesarean section (%)	66.7	60
5-min APGAR score	8.6 ± 1.1	8.8 ± 0.4
Need for resuscitation (%)	28.9	20
Risk of neonatal infection (%)	44.4	40
Respiratory distress syndrome (%)	73.3	100
Surfactant therapy (%)	48.9	60
Duration of mechanical ventilation (days)	4 ± 3	3 ± 2
Extubation performed ≥ 24 h and ≤ 48 h after birth (%)	44.5	80

Data are presented as mean (standard deviation) and percentage (%)

In preterm newborns who were successfully extubated, IDT and DE increased significantly at T2 and T3 compared with T1 (*p* < 0.05), with IDT peaking at T2, while EDT and DTF showed no significant changes across time (Table 2).

**Table 2:** Median and interquartile range (IQR) of diaphragmatic function measurements assessed by ultrasound at three distinct time points

T1 (during IMV)		T2 (during SBT)	T3 (during NIPPV)
Preterm newborns who were successfully extubated (*n* = 45)			
IDT (mm)	1.2 (1.0–1.5)^*^	1.4 (1.1–1.7)&	1.3 (1.1–1.6)
EDT (mm)	0.9 (0.6–1.2)	0.9 (0.7–1.2)	1.0 (0.8–1.1)
DTF (%)	44 (33–50)	43 (33–52)	45 (36–50)
DE (mm)	2.2 (1.9–2.6)^*^	2.6 (2.1–3.4)	2.4 (2.0–2.9)
Descriptive data of preterm newborns with extubation failure (*n* = 5)			
IDT (mm)	1.0 (0.9–2.3)	1.2 (1–2)	1.4 (1.1–2.1)
EDT (mm)	0.6 (0.5–1.4)	0.7 (0.6–1.3)	0.8 (0.7–1.2)
DTF (%)	66 (51–83)	66 (57–77)	66 (62–76)
DE (mm)	2.2 (1.9–2.9)	2.6 (1.8–3.2)	2.8 (1.9–3.4)

^*^p < 0.029 vs T2 and T3; ^&^*p* < 0.001 vs T3

*IDT*, inspiratory diaphragmatic thickness; *EDT*, expiratory diaphragmatic thickness; *DTF*, diaphragmatic thickening fraction; *DE*, diaphragmatic excursion

Preterm newborns with extubation failure showed a progressive increase in most diaphragmatic parameters, whereas DTF remained consistently elevated. Due to the small sample size (*n* = 5), no inferential analyses were performed; therefore, these results are presented descriptively in Table 2.

## Discussion

Only a limited number of studies have reported ultrasound-based reference values for diaphragmatic function in preterm newborns [9–13], and even fewer have specifically evaluated diaphragmatic function before extubation in this population [14].

In the present study, among preterm newborns who were successfully extubated, a significant increase in IDT was observed during the SBT (T2) and under NIPPV after extubation (T3) compared with values obtained during IMV with minimal settings (T1). This may reflect greater diaphragmatic muscle contraction during spontaneous inspiration at T2 and T3. No significant changes were found in EDT or DTF across the three time points. A significant increase in DE was observed during the SBT (T2) and under NIPPV (T3), compared to values during IMV with minimal settings (T1). As DE is associated with inspiratory volume, this increase may reflect greater respiratory effort by newborns to maintain adequate ventilation under lower inspiratory support during T2 and T3, in contrast to the assistance provided during IMV, even at minimal parameters. These findings suggest that the SBT increases respiratory effort without providing reliable indicators of extubation readiness in preterm newborns, consistent with previous reports [2, 4, 5].

Although no significant differences in DTF were observed across the three time points, values were high compared to reference ranges for spontaneously breathing healthy preterm newborns [11–13]. DTF, the ratio of IDT to EDT, reflects diaphragmatic response to inspiratory effort [6]. Elevated DTF in this study may be attributed to the use of narrow endotracheal tubes and the anatomical and physiological features of preterm infants—such as increased airway resistance, reduced lung compliance, high chest wall compliance, and immature respiratory muscles [1, 4], which are accentuated during invasive support and early post-extubation.

Assessment of diaphragmatic function by ultrasound may support more targeted clinical management strategies during the weaning and extubation process in newborns. Importantly, although all preterm newborns in this study successfully completed the spontaneous breathing trial (SBT), the increased respiratory effort observed during the SBT did not reliably predict extubation readiness and may have exposed these patients to unnecessary respiratory workload, potentially leading to clinical instability [14, 15].

In preterm newborns who experienced extubation failure, diaphragmatic ultrasound parameters showed a gradual increase across different ventilatory phases, which may reflect an adaptive response to changing respiratory demands. Diaphragmatic thickening fraction (DTF) remained consistently elevated at all evaluated time points, suggesting sustained diaphragmatic activation throughout the study period. Given the limited sample size, these findings should be interpreted with caution and are presented descriptively. Nevertheless, the observed patterns suggest that diaphragmatic ultrasound may provide complementary information on respiratory muscle behavior in preterm infants who fail extubation.

This single-center study limits external validity, although patient characteristics were comparable to those reported in similar neonatal intensive care unit populations. Ultrasound is an operator-dependent method, and reproducibility may vary across centers, equipment, and operator experience. In addition, the study sample included not only neonates with respiratory distress syndrome but also cases with heterogeneous lung disease and varying extubation timings, which may have influenced diaphragmatic function measurements. Finally, the small number of extubation failures (*n* = 5) precluded inferential analyses; nevertheless, these descriptive findings may provide preliminary reference values to support respiratory weaning and extubation assessment in preterm infants.

## Conclusion

In preterm newborns who underwent invasive mechanical ventilation and were successfully extubated, IDT and DE increased during the SBT and remained elevated after extubation, suggesting that the SBT may increase respiratory effort without reliably indicating extubation readiness in this population. Due to the small number of extubation failures, only descriptive analyses were performed. The diaphragmatic ultrasound findings described in this study support its use as a complementary, noninvasive tool to guide extubation readiness and to monitor post-extubation adaptation in preterm newborns.

## Supplementary Information

Below is the link to the electronic supplementary material.

ESM 1 (DOC 1.46 MB)

## Data Availability

Data will be made available on request to correpond ing author.

## Abbreviations

CPAP: Continuous positive airway pressure
DE: Diaphragmatic excursion
DTF: Diaphragmatic thickening fraction
EDT: Expiratory diaphragmatic thickness
IDT: Inspiratory diaphragmatic thickness
IMV: Invasive mechanical ventilation
NIPPV: Nasal intermittent positive pressure ventilation
PEEP: Positive end-expiratory pressure
SBT: Spontaneous breathing trial
