# Supplementary material for: Ultrasound assessment of diaphragmatic function during weaning and after extubation in preterm newborns: brief report
Source: Eur J Pediatr. 2026 Mar 25;185(4):211. doi: 10.1007/s00431-026-06866-x (PMC13017974; doi:10.1007/s00431-026-06866-x)
Supplement: Supplementary file 1 — (DOC 1.46 MB) [file 431_2026_6866_MOESM1_ESM.doc]

**Methods**

**Inclusion and Exclusion Criteria**

Preterm newborns who were intubated and received invasive mechanical ventilation (IMV) for at least 24 hours during the study period were included. Eligibility required clinical stability and readiness for a first extubation attempt as determined by the medical team, based on clinical evaluation and minimal ventilatory parameters. Exclusion criteria included congenital heart disease with hemodynamic significance, chest wall deformities, major congenital anomalies, chromosomal abnormalities, neurological disorders (e.g., hypoxic-ischemic encephalopathy), or intracranial hemorrhage greater than grade III. Newborns were also excluded if an intercurrent event prevented completion of the ultrasound assessment, if clinical or hemodynamic instability contraindicated extubation.

**Clinical features**

Sociodemographic and clinical data were collected to characterize the sample, including gestational age and weight at extubation, sex, ventilator settings, cardiorespiratory variables (heart rate, respiratory rate, oxygen saturation), prenatal steroid administration, mode of delivery (cesarean section), 5-minute APGAR score, need for resuscitation, risk of neonatal infection, presence of respiratory distress syndrome, and duration of mechanical ventilation. All participants subsequently underwent diaphragmatic ultrasound assessments.

**Extubation Criteria**

Extubation was indicated by the medical team based on unit-defined criteria, including satisfactory blood gases (no acid–base disturbances) and use of pressure assist-control ventilation, time-cycled, with peak inspiratory pressure <20 cmH₂O, expiratory tidal volume 4–6 mL/kg, and trigger sensitivity allowing spontaneous efforts. Other parameters included inspiratory time of 0.3–0.5 s, positive end-expiratory pressure (PEEP) of 5 cmH₂O, respiratory rate ≤20 breaths/min, spontaneous rate above the set rate, and inspired oxygen fraction (FiO₂) ≤40% with oxygen saturation >90%. Newborns also had to be clinically and hemodynamically stable, including those on low-dose vasoactive drugs, with no sedation for ≥6 hours before extubation. Methylxanthine use was at the team's discretion. Endotracheal suctioning was performed one hour before the SBT, following unit protocols.

**SBT**
During the SBT, intubated neonates were maintained on endotracheal CPAP with a PEEP of 5 cmH₂O, without additional pressure support, and with the same FiO₂ used during invasive ventilation, for at least 5 minutes. SBT failure, defined as bradycardia (<100 beats/min for ≥15 seconds) and/or SpO₂ <85% despite a 15% increase in FiO₂ [9], led to reconnection to mechanical ventilation and exclusion from the study if it prevented the ultrasound examination.

**Ultrasound Assessment of Diaphragm Thickness, Thickening Fraction, and Excursion**

Images for measurement were acquired using the frame-freeze function during periods of quiet breathing, in which the examiner identified a sequence of regular respiratory cycles with minimal variation in amplitude and consistent inspiratory diaphragmatic contractions. This method enabled more accurate assessment of diaphragmatic excursion and thickness. Diaphragmatic thickness and excursion were quantified using ImageJ software. To minimize measurement bias, the average of three consecutive respiratory cycles was used for all analyses.

**Thickness and Thickening Fraction**

Ultrasound evaluation of IDT and EDT phases of the respiratory cycle was performed in the zone of apposition of the right hemidiaphragm. This region was located along the mid-axillary line, between the 8th and 9th intercostal spaces, with the linear transducer positioned perpendicular to the ribs. Imaging was conducted in B-mode, and measurements were obtained in M-mode, specifically between the diaphragmatic pleura and the peritoneum at the end of both inspiration and expiration (FIG 1A and 1B). The DTF was calculated using the following formula: **DTF (%) = [(IDT – EDT) / EDT] × 100** [10].

**Excursion**

With the patient in the supine position, the transducer was placed in the anterior subcostal region and oriented cranially to ensure that the ultrasound beam was perpendicular to the posterior third of the right hemidiaphragm. B-mode imaging was used for anatomical localization, and DE was measured using M-mode. Excursion was defined as the difference in the position of the outer diaphragm line between peak inspiration and end expiration (FIG. 1C and 1D) [15].


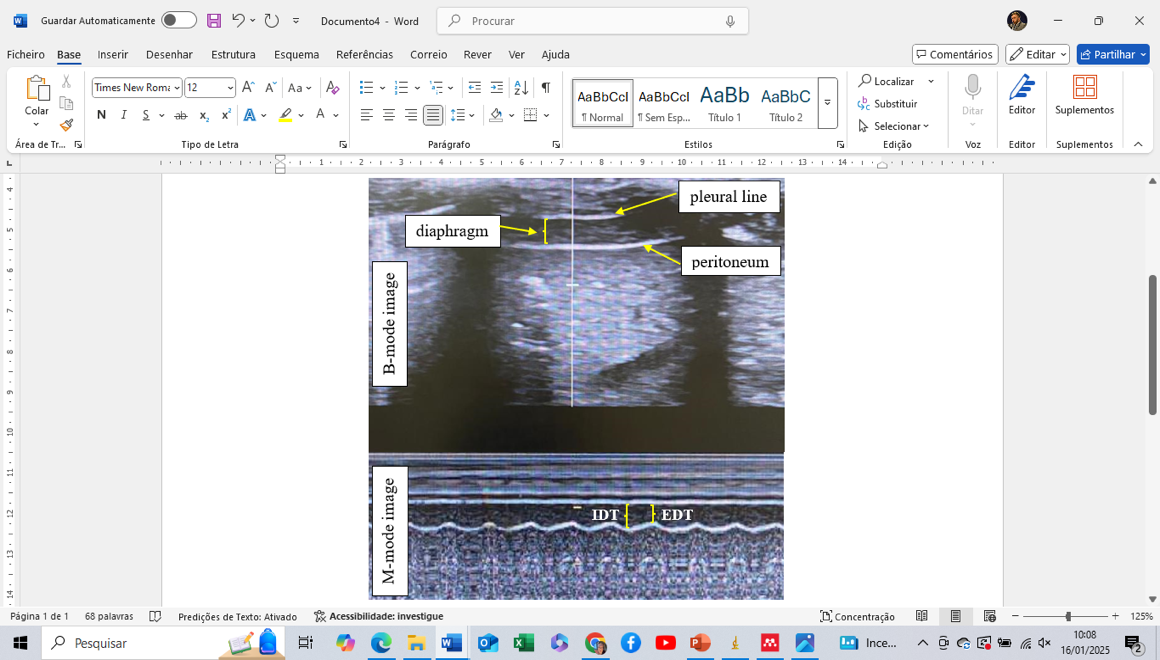

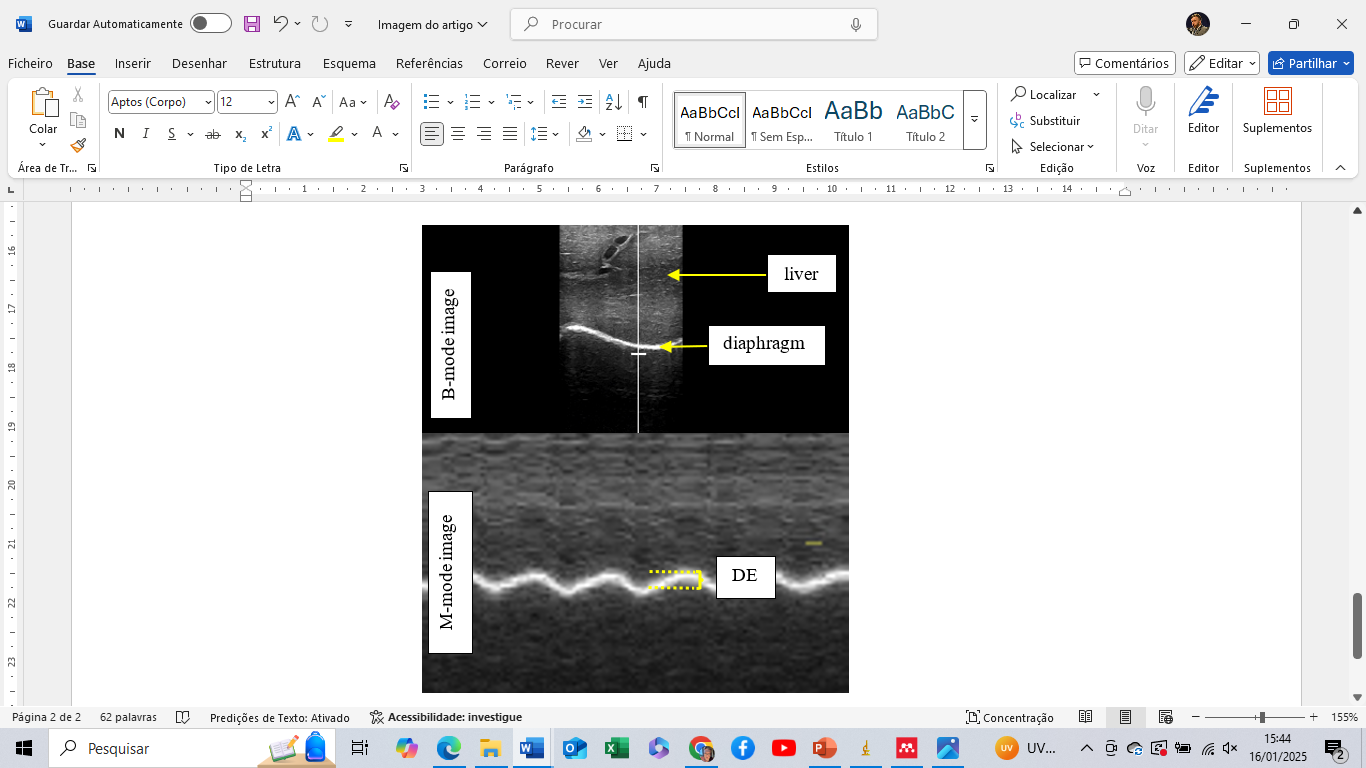

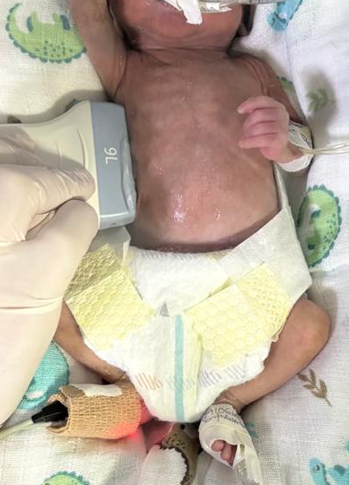

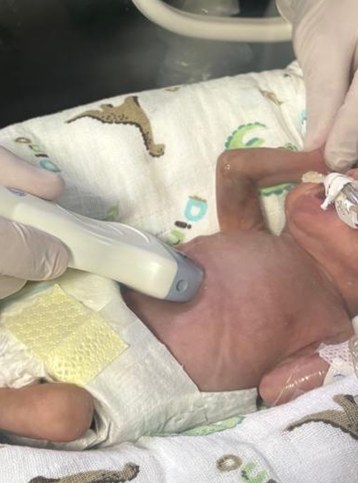


**B**

**A**

**C**

**D**

**Figure 1. Ultrasound Assessment of Diaphragmatic Thickness and Excursion
A and B** – Thickness Assessment: In B-mode, the diaphragm appears between the upper hyperechoic pleural line and the lower peritoneal line. M-mode shows the respiratory cycle, allowing measurement of IDT and EDT. **C and D** – Excursion Assessment: In B-mode, the liver acts as an acoustic window, with the diaphragm seen as a hyperechoic line. M-mode displays diaphragmatic motion of the right hemidiaphragm, enabling measurement of DE. Parental consent for publication of the images was obtained.

**Extubation Failure Criteria**

Extubation failure was defined, according to unit protocol, as the need for reintubation within the first 48 hours after removal of the endotracheal tube, based on the following criteria: (a) more than six episodes of apnea requiring stimulation within six hours, or more than one significant apnea episode requiring manual ventilation; (b) respiratory acidosis (PaCO₂ > 65 mmHg and pH < 7.25); (c) FiO₂ > 60% to maintain SpO₂ between 90 and 94% [9].

**Table 1.** Demographic characteristics of preterm newborns participating in the study (n = 50)

| **Variable** | | S**uccessfully extubated**  **n = 45** | **Extubation failure**  **n = 5** |
| --- | --- | --- | --- |
| **Mechanical Ventilation Parameters at study** | |  |  |
|  | Assist-control mode (n) | 45 | 5 |
|  | PIP (cmH_2_O) | 14 ± 2 | 15 ± 2 |
|  | VT (ml/Kg) | 5.5 ± 0.7 | 4.8 ± 0.8 |
|  | PEEP (cmH_2_O) | 5.4 ± 0.7 | 5.2 ± 0.4 |
|  | FiO_2_ (%) | 27 ± 3 | 27 ± 3 |
|  | Flow rate (l/min) | 7.0 ± 0.7 | 7.0 ± 0.5 |
|  | Ti (seconds) | 0.35 ± 0.03 | 0.36 ± 0.04 |
|  | MAP (cmH_2_O) | 7.2 ± 0.5 | 6.8 ± 0.4 |
|  | Mandatory respiratory rate (breaths/min) | 21 ± 2 | 22 ± 3 |

PIP, peak inspiratory pressure; VT, Exhaled tidal volume; PEEP, positive end-expiratory pressure; FiO_2_, fraction of inspired oxygen; Ti, inspiratory time; MAP, mean airway pressure.
